# MEK Inhibition in a Newborn with *RAF1*-Associated Noonan Syndrome Ameliorates Hypertrophic Cardiomyopathy but Is Insufficient to Revert Pulmonary Vascular Disease

**DOI:** 10.3390/genes13010006

**Published:** 2021-12-21

**Authors:** Alessandro Mussa, Diana Carli, Elisa Giorgio, Anna Maria Villar, Simona Cardaropoli, Caterina Carbonara, Maria Francesca Campagnoli, Paolo Galletto, Martina Palumbo, Simone Olivieri, Claudio Isella, Gregor Andelfinger, Marco Tartaglia, Giovanni Botta, Alfredo Brusco, Enzo Medico, Giovanni Battista Ferrero

**Affiliations:** 1 Department of Public Health and Pediatric Sciences, University of Torino, 10126 Torino, Italy; alessandro.mussa@unito.it (A.M.); diana.carli@unito.it (D.C.); simona.cardaropoli@unito.it (S.C.); simone.olivieri343@edu.unito.it (S.O.); 2 Clinical Pediatric Genetics Unit, Regina Margherita Children’s Hospital, Città Della Salute e Della Scienza di Torino, 10126 Torino, Italy; 3 Pediatric Onco-Hematology, Stem Cell Transplantation and Cell Therapy Division, Regina Margherita Children’s Hospital, Città Della Salute e Della Scienza di Torino, 10126 Torino, Italy; 4 Department of Molecular Medicine, University of Pavia, 27100 Pavia, Italy; elisa.giorgio@unipv.it; 5 IRCCS Mondino Foundation, 27100 Pavia, Italy; 6 Pediatric Cardiology Unit, Regina Margherita Children’s Hospital, Città Della Salute e Della Scienza di Torino, 10126 Torino, Italy; avillar@cittadellasalute.to.it; 7 Neonatal Intensive Care Unit, Sant’Anna Hospital, Città Della Salute e Della Scienza di Torino, 10126 Torino, Italy; ccarbonara@cittadellasalute.to.it (C.C.); macampagnoli@cittadellasalute.to.it (M.F.C.); pgalletto@cittadellasalute.to.it (P.G.); 8 Laboratory of Oncogenomics, Candiolo Cancer Institute, FPO-IRCCS, 10060 Candiolo, Italy; martina.palumbo@ircc.it (M.P.); claudio.isella@ircc.it (C.I.); enzo.medico@unito.it (E.M.); 9 Department of Oncology, University of Torino, 10126 Torino, Italy; 10 Cardiovascular Genetics, Department of Pediatrics, CHU Sainte Justine, Université de Montréal, Montreal, QC H3T 1C5, Canada; gregor.andelfinger@recherche-ste-justine.qc.ca; 11 Genetics and Rare Diseases Research Division, Ospedale Pediatrico Bambino Gesù, IRCCS, 00146 Rome, Italy; marco.tartaglia@opbg.net; 12 Department of Pathology, Sant’Anna Hospital, Città Della Salute e Della Scienza di Torino, 10126 Torino, Italy; giovanni.botta@unito.it; 13 Department of Medical Sciences, University of Torino, 10126 Torino, Italy; alfredo.brusco@unito.it; 14 Unit of Medical Genetics, Città Della Salute e Della Scienza di Torino, 10126 Torino, Italy; 15 Department of Clinical and Biological Sciences, University of Torino, 10043 Orbassano, Italy

**Keywords:** therapeutics, hypertrophic cardiomyopathy, Noonan syndrome, MEK-inhibitor, RASopathies, transcriptomics

## Abstract

The *RAF1*:p.Ser257Leu variant is associated with severe Noonan syndrome (NS), progressive hypertrophic cardiomyopathy (HCM), and pulmonary hypertension. Trametinib, a MEK-inhibitor approved for treatment of RAS/MAPK-mutated cancers, is an emerging treatment option for HCM in NS. We report a patient with NS and HCM, treated with Trametinib and documented by global RNA sequencing before and during treatment to define transcriptional effects of MEK-inhibition. A preterm infant with HCM carrying the *RAF1*:p.Ser257Leu variant, rapidly developed severe congestive heart failure (CHF) unresponsive to standard treatments. Trametinib was introduced (0.022 mg/kg/day) with prompt clinical improvement and subsequent amelioration of HCM at ultrasound. The appearance of pulmonary artery aneurysm and pulmonary hypertension contributed to a rapid worsening after ventriculoperitoneal shunt device placement for posthemorrhagic hydrocephalus: she deceased for untreatable CHF at 3 months of age. Autopsy showed severe obstructive HCM, pulmonary artery dilation, disarrayed pulmonary vascular anatomy consistent with pulmonary capillary hemangiomatosis. Transcriptome across treatment, highlighted robust transcriptional changes induced by MEK-inhibition. Our findings highlight a previously unappreciated connection between pulmonary vascular disease and the severe outcome already reported in patients with *RAF1*-associated NS. While MEK-inhibition appears a promising therapeutic option for HCM in RASopathies, it appears insufficient to revert pulmonary hypertension.

## 1. Introduction

Noonan syndrome (NS) is an autosomal dominant disorder affecting development and growth with a prevalence of 1 in 2000 live births. Major clinical features of the disorder include cardiac defects, mostly pulmonary valve stenosis and hypertrophic cardiomyopathy (HCM), postnatal short stature and failure to thrive, distinctive gestalt, cryptorchidism, skeletal and lymphatic anomalies, variable neurodevelopmental involvement, and bleeding diathesis [1]. NS is the most common among the RASopathies, a clinically variable family group of genetic conditions characterized by overlapping features and caused by upregulation of intracellular signalling through the Ras/mitogen-activated protein kinase (RAS-MAPK) pathway [2]. Specific genotype/phenotype correlations have been described in NS. Patients harboring pathogenic *PTPN11* or *SOS1* variants have a low prevalence of hypertrophic cardiomyopathy [3], while those with disease-causing *RAF1, RIT1* and *MRAS* variants are expected to develop this cardiac defect (~85%, 70%, and 100%, respectively) [4,5,6], showing the strongest association with HCM among RASopathies [7]. *RAF1* variants represent approximately 5% of the gene defects in NS, but account for many of the most severe cases [8]. Notably, the *RAF1*(NM_002880):c.770C>T (p.Ser257Leu) variant is associated with a severe clinical phenotype of NS with neonatal HCM and pulmonary hypertension [4,9]. Early-onset HCM represents the major determinant in the outcome of patients with RASopathies and is associated with cardiac failure, worse outcome and high risk of cardiac death [7,8,10].

Recently, precision medicine offered interesting treatment opportunities in the RASopathies. The RAS-MAPK pathway has a well-defined role in cancer biology and has been extensively studied as an important target in the development of targeted therapies. Several small-molecule inhibitors targeting the mitogen-activated protein/extracellular signal-regulated kinases 1 and 2 (MEK1 and MEK2), which are key signal transducers of the MAPK cascade, have been developed. Reducing the perturbed signal transduction of the RAS/MAPK pathway has been presented as promising for RASopathy-associated cardiomyopathies [7]. The use of a MEK inhibitor (MEKi) in a murine model of *RAF1*-related NS showed encouraging results, since inhibition of MEK was able to revert multiple features of the disease, including HCM [11]. Furthermore, Trametinib, a selective reversible inhibitor of MEK1/2 activity approved for treatment of cancers with RAS/MAPK pathway activation, was successfully employed in two *RIT1*-mutated newborns with NS and severe HCM, inducing reversal of cardiac failure and valvular obstruction [12].

Here, we report on a *RAF1*-mutated patient with severe NS, including neonatal HCM and pulmonary hypertension, treated with selective MEK inhibition by Trametinib.

## 2. Materials and Methods

### 2.1. RNA Extraction

Blood samples from the patient have been collected in sodium-heparin tubes at days 0, 7, 16, 30, and 37 of Trametinib treatment. Peripheral blood mononuclear cells (PBMCs) have been isolated by Ficoll-Hypaque density gradient centrifugation (Histopaque-1077, Sigma-Aldrich, St. Louis, MO, USA). Total RNA was extracted from PBMCs preserved in TRI Reagent using the Direct-zol RNA miniprep kit (Zymo Research, Irvine, CA, USA), according to the manufacturer’s instructions. RNA concentration was quantified using the Qubit Fluorometer (Thermo Fisher Scientific, Waltham, MA, USA). RNA quality was assessed by verifying the RNA integrity number (RIN) and percentage of RNA fragments > 200 nucleotides in size (DV200) with Agilent RNA Kits on a Bioanalyzer 2100 (Agilent, Santa Clara, CA, USA).

### 2.2. RNA Sequencing

Total RNA was processed for RNA-seq analysis with the TruSeq RNA Library Prep Kit v2 (Illumina, San Diego, CA, USA) following manufacturer’s instructions. Furthermore, it is quantified using the Qubit Fluorometer (Thermo Fisher Scientific). Library correct size and purity were checked on a Bioanalyzer 2100 (Agilent), using Agilent DNA High Sensitivity kit. Libraries were sequenced on a NextSeq 500 system (Illumina).

### 2.3. Gene Expression Quantification

Each fastq file was aligned using STAR [13] to version HG38 of the human genome. Gencode 27 was used as the transcriptome reference database and gene quantification was performed with feature counts [14]. To avoid noise from low-detection genes, genes not reaching 4 counts in at least one sample were removed, and a thresholding was applied by assigning a random value between 3 and 4 counts to values below 4 counts. The final processing step was trimmed mean of M values (TMM) normalization using the EdgeR package [15].

### 2.4. Gene Set Enrichment Analysis

The two samples at Day 7 were averaged, then for each gene the log2 ratio vs Day 0 was calculated at each time point. For each time point, log2ratio values were used to rank genes for pre-ranked GSEA, using standard parameters [16,17]. Log2 ratio between average expression at all treatment points vs Day 0 was also calculated and used for pre-ranked GSEA. To identify a core set of representative genes for the various modulated pathways, we selected genes that were in the GSEA leading edge of the respective signature at all time points. Among these, we further prioritized MKI67, a known reporter of cell proliferation [18], and genes potentially involved in cardiomyocyte physiopathology based on literature search.

## 3. Results

### 3.1. Clinical Report

A female preterm infant was born at 33 + 6 weeks of gestation, after a pregnancy characterized by polyhydramnios (amniotic fluid index at 31 + 2 weeks of gestation 133 mm) and HCM (septal thickness 9 mm, thoracic index 0.5), from healthy and non-consanguineous parents. Prenatal measurement of nuchal translucency, biochemical pregnancy screening and foetal biometry were normal. Apgar score was 3/5 at 1 and 5 minutes, respectively, with poor initiation of breathing, cyanosis, hypotonia and hyporeflexia. The patient required urgent intubation and mechanical ventilation. Neonatal weight was 2150 g (+0.4 SDS), length 49.0 cm (+2.4 SDS), OFC 32.1 cm (+0.9 SDS). Clinical features were dominated by facial and nuchal dysmorphisms, highly suggestive for NS.

Echocardiography, performed shortly after birth, confirmed severe biventricular obstructive HCM with minimal left intraventricular gradient, mild mitral regurgitation, aortic valve with thickened cusps and dysplastic pulmonary valve with normal origin and caliber of the pulmonary branches. No direct or indirect signs of pulmonary hypertension were present and β-blockade treatment with propranolol was started.

At the 2nd day of life, she underwent a routine transfontanellar ultrasound demonstrating a grade II cerebral ventricular hemorrhage. Blood tests, including clotting tests, were normal. On blood extracted DNA, a next generation sequencing gene panel targeted to the RASopathy genes revealed a de novo c.770C>T (p.Ser257Leu) pathogenic variant in *RAF1* (NM_002880), confirming the clinical diagnosis of NS.

In the next days, she progressively developed severe congestive heart failure (CHF) characterized by profound hypoxic crises with moderate hypercarbia and requiring incremental ventilator support with high-frequency oscillatory ventilation and CHF drugs introduction (furosemide and spironolactone). She progressively developed a post-hemorrhagic hydrocephalus and an external ventricular drain was placed at 21 days subsequently replaced at 39 days of life. In the next week, her ventilatory and inotrope requirements continuously increased. Given the near-terminal CHF, having no other treatment options besides cardiac transplant, Trametinib was introduced (0.022 mg/kg/day) as an off-label prescription with parents’ consent and under approval of the local Ethical Committee for off-label prescriptions (47th day of life, treatment day +0).

After treatment initiation there was a prompt improvement in clinical conditions (ROSS score from IV to III), allowing progressive and rapid withdrawal of inotropes in the next 4 days and weaning from mechanical ventilation a week later. Consistent with HCM stabilization, nt-pro-BNP decreased from 30,805 to 2355 pg/mL, and the patient was placed in non-invasive ventilator support and restricted fluid intake, with furosemide administration “as needed”. During the next month, treatment with Trametinib was continued without relevant side effects. ROSS score was II at 1 month. Liver enzymes, complete blood count, clotting tests, electrolytes, renal function were regularly monitored with no substantial modifications from baseline nor effects attributable to Trametinib administration. Echocardiography showed a tendency to HCM improvement with reduction of the septal thickness from treatment start to day +23. We also noted sudden dilation of the pulmonary artery dilation (14 mm, +3.5 SDS) at day 23. Despite of this finding, clinical conditions were stable in the following days and non-invasive ventilatory support was progressively reduced in terms of oxygen flow and fraction.

**Figure 1:**
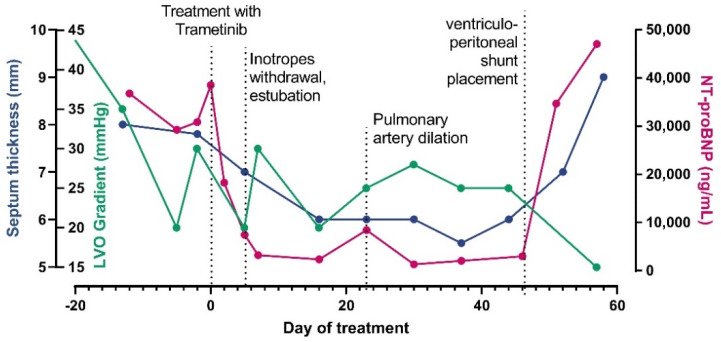
Treatment time course. The picture shows changes in nt-pro-BNP, Left Ventricular Outflow (LVO) gradient and septal thickness at ultrasound before and after treatment initiation and related to the major clinical events in our patient.

**Figure 2:**
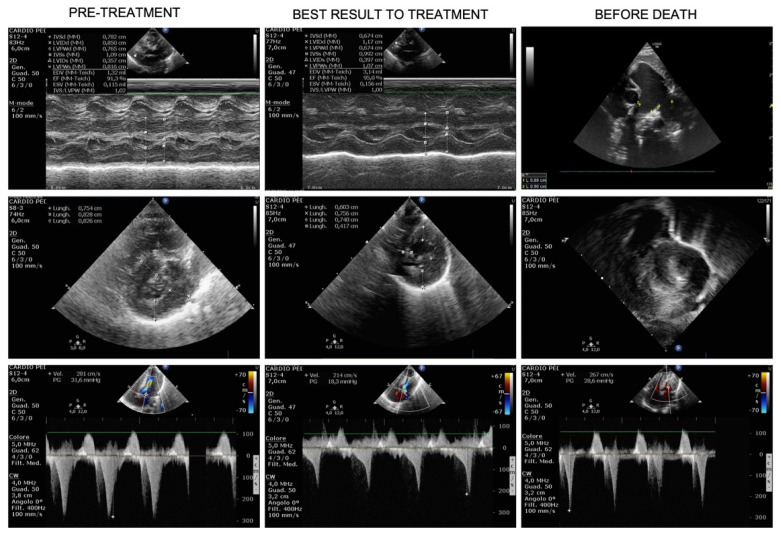
Evolution over time of hypertrophic cardiomyopathy (HCM). Pre-treatment US (first column) shows severe HCM at M-mode Left Ventricle (Mm-LV, first row), short-axis LV (second row) and LV outflow track obstruction (CW-Doppler, third row), few days before treatment start. The best result under treatment is shown in the middle column: Mm-LV in first row, short-axis LV (second row) and LV outflow track obstruction (third row) demonstrated a consistent reduction of LV thickness and improvement of LV outflow track obstruction. The third column shows cardiac US three days before death: pulmonary artery dilatation is evident in the first row (2D), the rapid worsening of HCM, both in terms of LV thickness and obstruction are depicted in the last 2 rows (short-axis LV and CW-Doppler, respectively).

At day +46, a ventriculoperitoneal shunt was placed in order to manage the intraventricular hemorrhage and a red blood cell unit was transfused. After surgery, we observed rapid worsening of CHF (nt-pro-BNP increased from 2049 pg/mL to 47,000 pg/mL) as well as respiratory deterioration, requiring again mechanical ventilator support. Pulmonary artery dilation, stable until then, increased to 18 mm (+5.7 SDS) and death from untreatable CHF with hypoxic respiratory failure occurred on day +57. Figure 1 recapitulates the time course with septal thickness changes, left ventricular outflow (LVO) gradient and nt-pro-BNP changes across treatment. Figure 2 shows the ultrasound HCM evolution over time.

**Figure 3:**
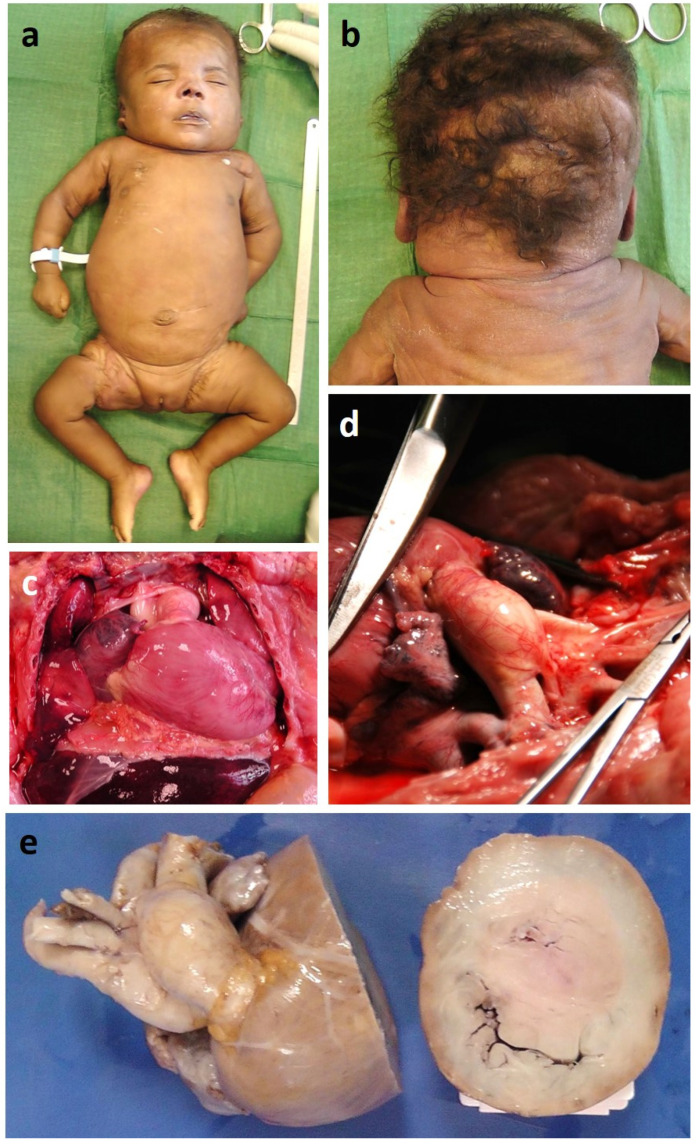
Gross pathology findings. Autopsy showed typical Noonan syndrome (NS) features with low-set posteriorly rotated ears, high anterior hairline with wide forehead and narrow temples, mild hypertelorism, downslanting palpebral fissures, broad nose, full tip with deeply grooved philtrum (**a**), pterigium colli, short webbed neck (**b**), severe obstructive HCM (**c**,**e**), dilation of pulmonary artery trunk and branches (**d**).

**Figure 4:**
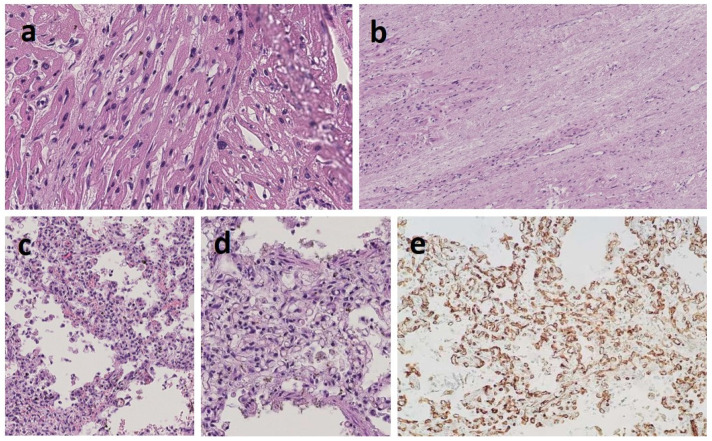
Histology showed a complete disarray of both gross and fine cardiac and pulmonary anatomy. Heart microscopy showed disorientation of muscle fibers and hypertrophic myocells with enlarged ovoid nucleus (**a**), left ventricle fibrotic and necrotic areas of myocells (long-standing infarction) and limited neighboring areas of more recent ischemic damage (**b**). Picture of the lungs with arterial hypertension, chronic stasis and alveolar damage: pulmonary capillary hemangiomatosis with peripheral arterioles with stenotic lumen for muscular tunic hypertrophy, intimal tunic thickening, hilar pulmonary vessels of increased caliber and with hypertrophic wall, thickened and hypervascularized interalveolar septa, alveolar lumens with exudate and foamy histiocytes containing hemosiderin (**c**), and increased thickness of the interalveolar septum with hypercapillarization (**d**). The capillaries (colored in brown with immunohistochemical staining CD31) were markedly increased in with a chaotic distribution (hemangiomatosis) with most of them not reaching the alveolar surface (**e**).

Autopsy confirmed a severe obstructive HCM as well as dilation of pulmonary artery trunk and branches (Figure 3). Pulmonary histology showed a complete disarray of both gross and fine pulmonary vascular anatomy consistent with diffuse blood congestion and alveolar damage, alveolar lumens with exudate and foamy histiocytes containing hemosiderin, septal fibrosis, thick-walled pulmonary arteries, thickened inter-alveolar septum with proliferation of blood vessels and capillaries randomly arranged and spaced apart from the alveolar lumen (Figure 4). The histologic features were consistent with an aberrant and excessive capillary proliferation and layering consistent with a diagnosis of pulmonary capillary hemangiomatosis.

### 3.2. Transcriptomics

**Figure 5:**
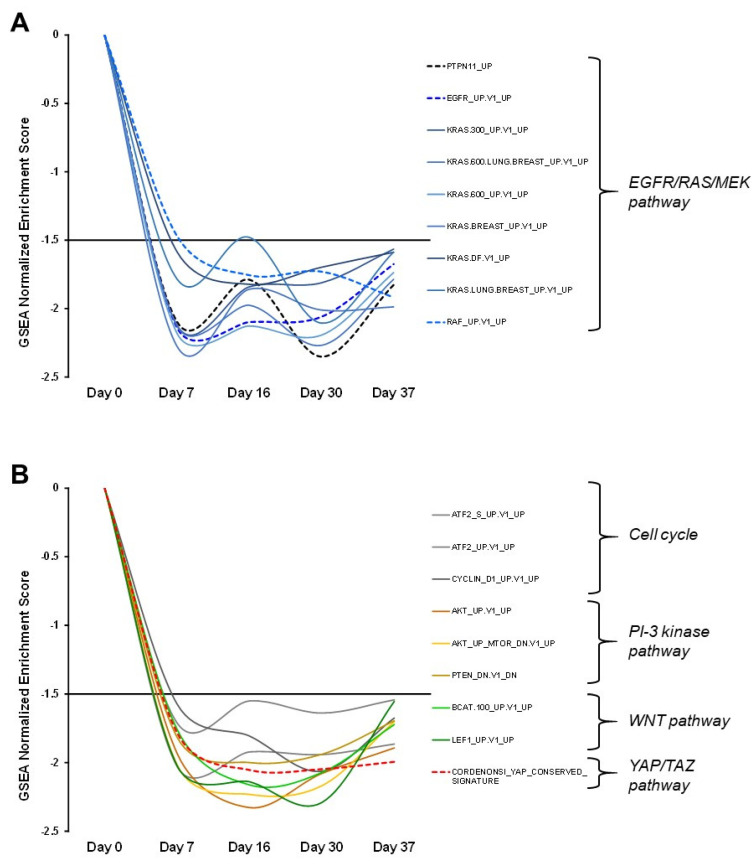
GSEA analysis of trametinib-driven pathway signature dynamics in the PBMC transcriptome. All signatures display a consistently negative Enrichment Score, below the −1.5 significance threshold value, indicating overall down-modulation by trametinib over the entire treatment period. (**A**) Modulation of signatures related to the EGFR/RAS/RAF/MEK pathway. These include the PTPN11 RASopathy signature, highlighted by the black dashed line. (**B**) Modulation of signatures associated with additional pathways, including the YAP/TAZ pathway signature highlighted by the red dashed line.

Peripheral blood mononuclear cells (PBMCs) were obtained at days 0, 7, 16, 30, and 37 of Trametinib treatment. Gene expression analysis was performed by global mRNA sequencing, and revealed marked changes in the transcriptional profiles, already apparent at day 7 and generally sustained over the treatment course. To systematically explore functional pathway alterations driven by Trametinib, we employed GeneSet Enrichment Analysis (GSEA) [16,17], which revealed sustained downregulation of many gene signatures, excluding the occurrence of adaptive response to the treatment (Supplementary Table S1). A particularly large group of signatures was associated with RAS/MAPK signaling (Figure 5A), including EGFR, RAS, RAF, and the previously published signature previously characterized in PBMCs from NS patients with mutated *PTPN11* alleles [19]. Further signatures associated with related pathways were also persistently downregulated, including cell cycle, PI-3 kinase, WNT and YAP/TAZ pathways (Figure 5B). In-depth analysis of representative genes for these pathways highlighted key transcriptional targets of trametinib potentially involved in heart and lung tissue homeostasis (Supplementary Figure S1, Table S2).

## 4. Discussion

Molecular therapy for congenital defects represents one of the most interesting and innovative advancements of precision medicine [20]. RASopathies represent a paradigmatic group of genetic diseases that could benefit from these innovative therapeutic approaches. In a newborn affected by a severe phenotype of NS with progressive HCM and pulmonary hypertension, due to a *RAF1* p.Ser257Leu variant, we reasoned that MEK inhibition might limit the progression of cardiac disease. This assumption relied on the observation of phenotype reversal under MEK inhibition of murine models of severe NS [11] and on the encouraging results obtained in *RIT1*-mutated NS patients with HCM [12]. MEK inhibitor treatment was initially characterized by a rapid and significant improvement in the overall clinical conditions of the patient, associated with a relevant nt-pro-BNP decrease consistent with Trametinib introduction. A clear response to the treatment was observed after a previous progressive negative evolution of the cardiac and pulmonary picture since birth that was evolving to a terminal CHF.

RAS activation is involved in the physiologic and pathologic cardiac hypertrophy, and the p.Ser257Leu and p.Leu613Val pathogenic variants have extensively been studied for their severe impact on myocardial physiology [21,22]. Indeed, the p.Ser257Leu variant in *RAF1* has been causally associated with severe pulmonary arterial hypertension [23] and progressive HCM [24] with lethal outcome. Several reports link this variant to a particularly severe NS phenotype [4,25,26,27,28], and activating *RAF1* variants are well known to be highly correlated with HCM [4,7,8,25,29,30,31]. The patient we describe presented a very severe phenotype complicated by prematurity. In addition to progressive HCM, she finally developed pulmonary hypertension, a clinical feature described in several patients with the p.Ser257Leu amino acid substitution in *RAF1* [24] and hydrocephalus, also already reported in association with this variant [24], in this case likely related to intraventricular hemorrhage of prematurity. Pulmonary artery dilation, consistent with an aneurysm, has been rarely observed in NS, reported only in a single newborn and in an adult patient [32], whereas cardiac and great arteries aneurysms have been sporadically described as part of the NS phenotype [33,34,35,36,37,38]. The pulmonary artery dilation observed in this patient was likely an epiphenomenon of the pulmonary hypertension (PH) and the histological abnormalities observed in the lungs: histology revealed pulmonary capillary hemangiomatosis (PCH), a very rare cause of PH characterized by extensive proliferation of pulmonary capillaries within alveolar septae [39] never reported in NS. Despite frequent fatal outcomes in severe NS, few pulmonary autopsy reports have been described so far [40,41]. Our case suggests that one of the most prevalent and deleterious pathogenic *RAF1* variants is characterized by a unique histopathological pulmonary finding, clearly related to the lethal outcome in the context of a respiratory course. The pulmonary findings observed are intriguing, since pulmonary vascular bed remodeling characterizing PCH and carcinogenesis have analogous features, such as the altered crosstalk between cells form different types of tissues and proliferation of pulmonary smooth muscle and endothelial cells [42]. Moreover, MAPK signaling has been identified as a key player in heritable forms of pulmonary arterial hypertension [43], with a role in the control of vascular remodeling. In this case, we suggest that the recurrent postnatal hypoxic episodes played a synergistic effect on the genetic predisposition with hyperactivation of the RAS-MAPK pathway. Thus, hypoxia and CHF could have triggered pulmonary microvascular remodeling, inducing a negative feed-back loop which hesitated to respiratory failure and worsening of the CHF [44].

At the same time of the therapeutic attempt, we collected patient’s PBMC at different timepoints and performed whole RNA sequencing. We decided to use PBMC as surrogate target cells to study the effects that can occur in target organs, such as the heart, because PBMC can be easily and repeatedly collected and are suitable for studying expression changes over time. However, as a limitation of this approach, it is not known whether the changes in gene expression levels in PBMCs are a reliable indicator of changes in target organs. We observed marked changes in the transcriptome, with a strong and sustained downregulation of signatures related to the SHP2 and RAS/MAPK signaling [19], which can be used as surrogate markers to measure Trametinib effect.

Notably, the RAS/MAPK axis was not the only pathway found downmodulated. Further downregulated signatures included those associated with the PI3 kinase, WNT and YAP/TAZ pathways, indicating that MEK inhibition exerted a broad effect on signal transduction. The YAP/TAZ pathway was particularly interesting, because it has been shown to promote cardiomyocyte proliferation during development [45]. Indeed, RASopathy-driven YAP/TAZ hyperactivity could be related to the cardiac hypertrophy observed in the patient, potentially explaining the mechanism for the sudden response to treatment, through trametinib-driven YAP/TAZ downmodulation. Among the most consistently downregulated genes, HB-EGF and SPP1 have been linked to cardiac hypertrophy by ERK pathway activation [46]. THBS1, SPP1 and LGR1 were previously associated with cardiomyocyte fibrosis, and heart failure [47,48]. LMO2 is involved in angiogenesis and endothelial cell proliferation [49].

Several causes can be linked to the fatal outcome of our case. It may be conceivable that some sort of “honeymoon phenomenon” has occurred for the drug. However, we observed that the expression downregulation of the signatures was sustained along the entire treatment course, excluding the occurrence of adaptive response to the treatment. Rather, the negative evolution could be linked to secondary remodeling of the pulmonary vascular bed which could have led to a worsening of the pulmonary perfusion dynamics. Trametinib, despite being effective at the cardiac level, likely failed to control the pulmonary vascular component of the disease process. It is noteworthy that in the model of genetic forms of pulmonary hypertension-BMPR2 loss-of function-ERK1/2 are constitutively activated and two Raf inhibitors (Sorafenib and AZ628) as well as Nintedanib (a triple receptor tyrosine kinase inhibitor acting upstream) reversed the invasive proliferation of the pulmonary artery endothelial cells [43]. Imatinib, a tyrosine kinase inhibitor, has been observed to be efficient in reversing the cardiopulmonary remodeling in pulmonary hypertension, representing a potential candidate for a combined therapy [50], which has shown synergistic effects in cancer models [51].

The timing and rapidity of the terminal clinical worsening observed connects the deteriorating clinical condition with the surgery for ventriculoperitoneal shunt placement. Undoubtedly, the extremely precarious clinical conditions have been disturbed in their delicate balance by such concomitant factors complicating its management.

## 5. Conclusions

In our opinion, the prompt improvement and subsequent clinical stability that characterized our case from the moment of the introduction of the MEK inhibitor until the final rapidly evolving CHF makes this experience encouraging despite the outcome. The use of MEK inhibitors remains a promising therapeutic option for HCM in the RASopathies and further experience in this area, in patients with associated pulmonary hypertension, is needed. Finally, the unique histopathological pulmonary finding highlights the connection between pulmonary vascular disease and the severe clinical outcome generally associated with the *RAF1* p.Ser257Leu amino acid substitution, besides underlying the paucity of data on the pulmonary issues of RASopathy patients and suggesting that this should be looked at more frequently.

## Data Availability

The datasets generated and analyzed during the current study are available from the corresponding author on reasonable request.

## Supplementary Materials

The following are available online at https://www.mdpi.com/article/10.3390/genes13010006/s1, Figure S1: Pathway and gene modulation by trametinib in PBMCs. Table S1: Results of GeneSet Enrichment Analysis (GSEA) on “Oncogenic Signature Gene Sets” from the Molecular Signature Database, subset C6 (http://www.gsea-msigdb.org/gsea/msigdb/genesets.jsp?collection=C6, accessed on 7 September 2021) (a) and on the PTPN11-UP Noonan Signature (https://pubmed.ncbi.nlm.nih.gov/22253195/, accessed on 7 September 2021) (b), comparing different days of treatment vs pretreatment control., Table S2: Results of GSEA on “Oncogenic Signature Gene Sets” from the Molecular Signature Database with in-depth analysis of representative genes for selected pathways with key transcriptional targets of trametinib potentially involved in heart and lung tissue homeostasis.
