# Supplementary figures and images for: MEK Inhibition in a Newborn with RAF1-Associated Noonan Syndrome Ameliorates Hypertrophic Cardiomyopathy but Is Insufficient to Revert Pulmonary Vascular Disease
Source: Genes (Basel). 2021 Dec 21;13(1):6. doi: 10.3390/genes13010006 (PMC8774485; doi:10.3390/genes13010006)

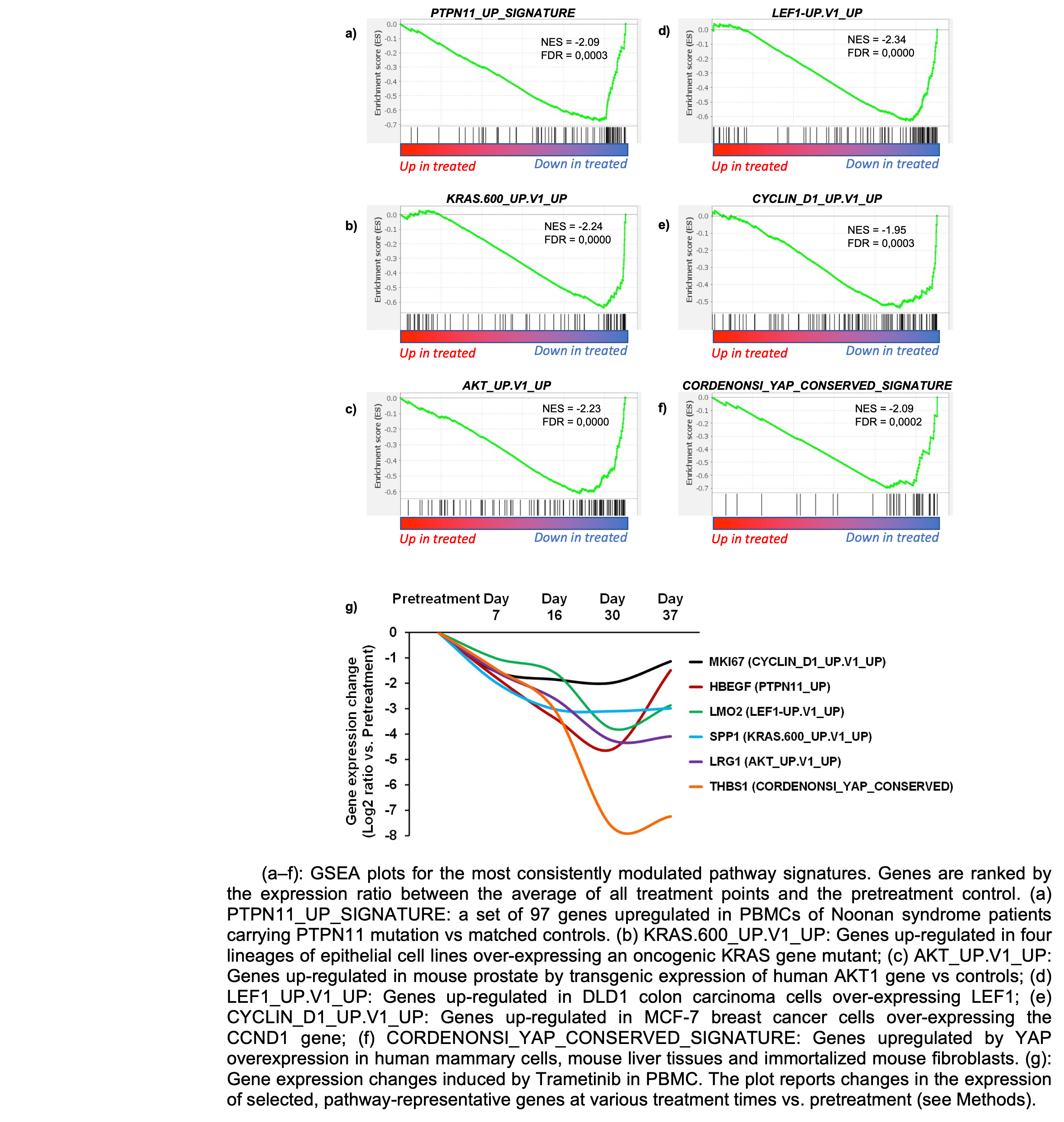

Supplement: Supplementary file 1 [file genes-13-00006-s001.zip › genes-1508422-supplementary/Figure S1.jpg]
